# Routine use of HbA1c amongst inpatients hospitalised with decompensated heart failure and the association of dysglycaemia with outcomes

**DOI:** 10.1038/s41598-018-31473-8

**Published:** 2018-09-10

**Authors:** K. Khoo, J. Lew, P. Neef, L. Kearney, L. Churilov, R. Robbins, A. Tan, M. Hachem, L. Owen-Jones, Q. Lam, G. K. Hart, A. Wilson, P. Sumithran, D. Johnson, P. M. Srivastava, O. Farouque, L. M. Burrell, J. D. Zajac, E. I. Ekinci

**Affiliations:** 1 Department of Endocrinology, Austin Health, Heidelberg, 3084 Victoria, Australia; 2 Department of Medicine, Austin Health, The University of Melbourne, Heidelberg, 3084 Victoria, Australia; 3 Department of General Medicine, Austin Health, Heidelberg, 3084 Victoria, Australia; 4 Department of Cardiology, Austin Health, Heidelberg, 3084 Victoria, Australia; 5 The Florey Institute of Neuroscience and Mental Health, Heidelberg, 3084 Victoria, Australia; 6 Department of Strategy, Quality & Service Redesign, Austin Health, Heidelberg, 3084 Victoria, Australia; 7 Austin Centre for Applied Clinical Informatics, Austin Health, Heidelberg, 3084 Victoria, Australia; 8 Department of Pathology, Austin Health, Heidelberg, 3084 Victoria, Australia; 9 Department of Intensive Care, Austin Health, Heidelberg, 3084 Victoria, Australia; 10 Health and Biomedical Informatics Centre, University of Melbourne, Parkville, 3010 Victoria, Australia; 11 St Vincent’s Hospital Melbourne, Fitzroy, 3065 Victoria, Australia

## Abstract

Diabetes is an independent risk factor for development of heart failure and has been associated with poor outcomes in these patients. The prevalence of diabetes continues to rise. Using routine HbA1c measurements on inpatients at a tertiary hospital, we aimed to investigate the prevalence of diabetes amongst patients hospitalised with decompensated heart failure and the association of dysglycaemia with hospital outcomes and mortality. 1191 heart failure admissions were identified and of these, 49% had diabetes (HbA1c ≥ 6.5%) and 34% had pre-diabetes (HbA1c 5.7–6.4%). Using a multivariable analysis adjusting for age, Charlson comorbidity score (excluding diabetes and age) and estimated glomerular filtration rate, diabetes was not associated with length of stay (LOS), Intensive Care Unit (ICU) admission or 28-day readmission. However, diabetes was associated with a lower risk of 6-month mortality. This finding was also supported using HbA1c as a continuous variable. The diabetes group were more likely to have diastolic dysfunction and to be on evidence-based cardiac medications. These observational data are hypothesis generating and possible explanations include that more diabetic patients were on medications that have proven mortality benefit or prevent cardiac remodelling, such as renin-angiotensin system antagonists, which may modulate the severity of heart failure and its consequences.

## Introduction

The prevalence of diabetes mellitus is rising and patients with diabetes are at increased risk of cardiovascular disease and its associated clinical complications. Diabetes is recognised as an independent risk factor for the development of heart failure^1^ and as a coronary heart disease equivalent. Heart failure and peripheral arterial disease are now the most common first presentations of cardiovascular disease in patients with diabetes, as the incidence of myocardial infarction and stroke has declined rapidly over the last few decades^2^. The relative risk of heart failure is doubled in men with diabetes and increased by five-fold in women with diabetes^3^. Independently of diabetes, heart failure prevalence is also rising and represents a significant public health issue due to its impact on quality of life and health expenditure with frequent hospital admissions^4^.

Previous studies have demonstrated that approximately 40% of patients with heart failure have diabetes^5–7^ and this prevalence is rising^4,8^. A recent study into acute heart failure admissions in Australia found that the prevalence of diabetes in these patients was 38%^9^. In these studies, methods of defining diabetes other than HbA1c testing were employed, such as fasting blood glucose levels or a previous diagnosis of diabetes, and therefore the true prevalence of diabetes was likely underestimated. The use of HbA1c is now recognised as a valid method for the diagnosis of diabetes^10,11^.

In patients with heart failure, concomitant diabetes has been found to have an independent association with all-cause mortality and with cardiovascular death^12,13^ as well as a higher hospital readmission rate^6^. This has been demonstrated in patients with both reduced and preserved ejection fraction^4,14^. However, patients with diabetes are also more likely to be prescribed cardioprotective medications, in particular ACE inhibitors and statins^4^.

The relationship between HbA1c and mortality in patients with diabetes and heart failure is insufficiently studied with conflicting results. Some cohort studies have demonstrated a higher risk of mortality with higher HbA1c^15^, whilst others have demonstrated a lower risk^16,17^ and some studies have demonstrated a U-shaped association^18,19^. A Scottish population study over a 17-year period (n = 116556) demonstrated that in patients with a new diagnosis of heart failure requiring a hospital admission, diabetes was significantly and independently associated with a lower mortality rate at 30 days but by one year, diabetes was an independent predictor of mortality, particularly in those less than 75 years of age^20^. It also demonstrated that diabetes independently predicted heart failure readmission. More recent studies utilising the European Society of Cardiology and Heart Failure Association heart failure long-term registry have also found that in both hospital inpatients with acute heart failure^21^ and in ambulatory patients with heart failure^22^, diabetes was independently associated with an increased risk of 1-year all-cause mortality, cardiovascular death and heart failure hospitalisation. Patients hospitalised with acute heart failure also had a higher risk of in-hospital mortality^21^.

Given these previous findings, we hypothesised that patients admitted with heart failure who also had a diagnosis of diabetes mellitus would have a higher readmission rate, worse hospital outcomes and a higher mortality rate compared to patients without diabetes mellitus.

Therefore, the aims of our study were to investigate the prevalence of diabetes mellitus amongst patients hospitalised with a diagnosis of heart failure using routine HbA1c testing on admission and to determine the association between dysglycaemia and patient outcomes.

## Results

**Figure 1:**
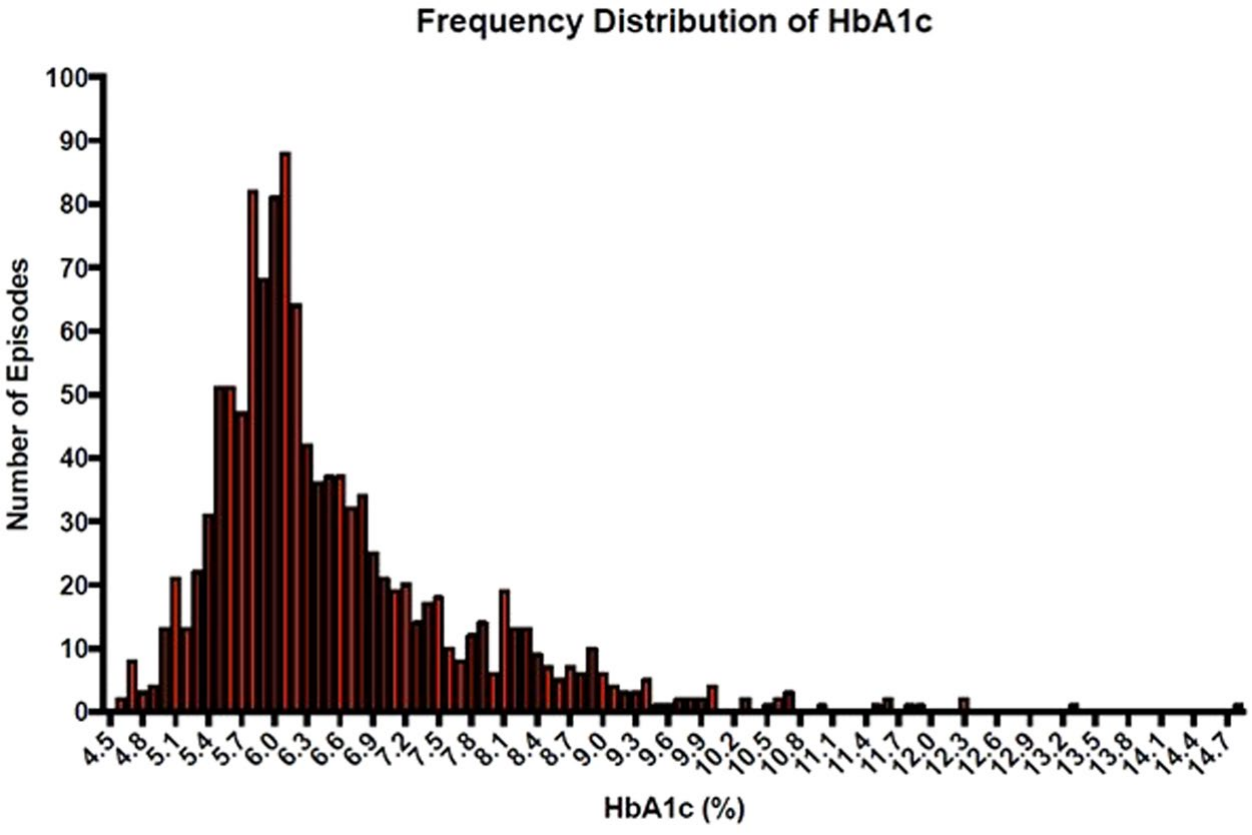
Frequency distribution of HbA1c.

1191 heart failure admission episodes with an HbA1c result within 90 days prior to or 7 days post-admission were identified. Of these admissions, 49% of the patients had diabetes, 34% had pre-diabetes and 17% did not have diabetes. Patients with diabetes were younger and there was a higher proportion of men in the diabetes group compared with those with pre-diabetes or without diabetes (Table 1). The median HbA1c for the diabetes group was 7.0% (53 mmol/mol) (Fig. 1). The median haemoglobin and CKD-EPI eGFR were highest in the pre-diabetes group and the median Charlson comorbidity score (excluding diabetes and age) was highest in the diabetes group.

**Table 1:** Patient characteristics by diabetes status.

Clinical characteristics	N (episodes)	Diabetes	Pre-diabetes	No diabetes	p value^¶^
Number	1191	581 (49%)	408 (34%)	202 (17%)	
Male (%)	1191	323 (55.6%)	206 (50.5%)	90 (44.6%)	0.02
Age (years)	1191	79 (72, 84)	84 (77, 88)	83 (74, 88)	0.0001
HbA1c (%)	1191	7.0 (6.5, 8.0)	6.0 (5.8, 6.1)	5.4 (5.2, 5.5)	0.0001
HbA1c (mmol/mol)	1191	53 (48, 64)	42 (40, 43)	36 (33, 37)	0.0001
Haemoglobin (g/L)	1187	117 (104, 131)	124 (111, 137)	114 (103, 129)	0.0001
eGFR* (CKD-EPI) (ml/min/1.73 m2)	1187	39 (24, 60)	47 (29, 66)	39 (18, 71)	0.0013
Charlson comorbidity score (excluding diabetes and age)^#^	1191	3 (1, 3)	1 (1, 2)	1 (1, 3)	0.0001

Categorical explanatory variables are reported as percentages and continuous explanatory variables are summarized as medians with interquartile ranges in parentheses.

^¶^*p-*values were determined by Fisher’s exact test for categorical variables and Kruskal-Wallis for continuous variables.

^†^p-value compared to group with an HbA1c within the pre-specified time frame. P-values were determined by Fisher’s exact test for categorical variables and Wilcoxon rank-sum test for continuous variables.

^#^Charlson comorbidity score - a validated method of weighting chronic medical conditions (the scores for diabetes and age were excluded as these are included as separate variables).

Abbreviations:* = eGFR, estimated glomerular filtration rate derived using the CKD-EPI (Chronic Kidney Disease-Epidemiology Collaboration) equation formula; HbA1c = glycosylated haemoglobin; ICU = Intensive Care Unit.

A further 270 heart failure admission episodes within the study period were identified which did not have the requisite HbA1c result to be included in our study population. There were no statistically significant differences in baseline characteristics between this group and those with an HbA1c result within the defined parameters (Table 2).

**Table 2:** Patient characteristics – Heart failure admissions without HbA1c.

Clinical characteristics	N (episodes)		p value^†^
Male (%)	270	156 (57.8%)	0.09
Age (years)	270	81 (75, 86)	0.66
Haemoglobin (g/L)	256	118 (103, 130)	0.44
eGFR* (CKD-EPI) (ml/min/1.73 m2)	255	44 (29, 62)	0.95
Charlson comorbidity score (excluding diabetes and age)^#^	270	1 (1, 3)	0.59

Categorical explanatory variables are reported as percentages and continuous explanatory variables are summarized as medians with interquartile ranges in parentheses.

^¶^*p-*values were determined by Fisher’s exact test for categorical variables and Kruskal-Wallis for continuous variables.

^†^p-value compared to group with an HbA1c within the pre-specified time frame. P-values were determined by Fisher’s exact test for categorical variables and Wilcoxon rank-sum test for continuous variables.

^#^Charlson comorbidity score - a validated method of weighting chronic medical conditions (the scores for diabetes and age were excluded as these are included as separate variables).

Abbreviations:* = eGFR, estimated glomerular filtration rate derived using the CKD-EPI (Chronic Kidney Disease-Epidemiology Collaboration) equation formula; HbA1c = glycosylated haemoglobin; ICU = Intensive Care Unit.

Of the 1191 heart failure admissions with an HbA1c result, 58% were admitted under a General Medical unit, 26% under the Cardiology unit and the remaining 16% under other specialty medical and surgical units. Patient outcomes by diabetes status are shown in Table 3. There were no significant differences between the diabetes, pre-diabetes and no diabetes groups in median length of stay, intensive care unit admission rates or 28-day readmission rate. Using a multivariable analysis adjusting for age, Charlson comorbidity score (excluding diabetes and age) and estimated glomerular filtration rate, there was no evidence of diabetes being associated with length of stay, ICU admission or 28-day readmission.

**Table 3:** Patient outcomes by diabetes status.

Outcome	N (episodes)	Diabetes	Pre-diabetes	No diabetes	p value^*¶*^
Length of stay (days, excluding HITH)	1191	5 (3, 8)	4 (2, 7)	4 (3, 8)	0.26
ICU admission (%)	1191	27 (4.6%)	12 (2.9%)	7 (3.5%)	0.39
Mechanical ventilation (%)	1191	6 (1.0%)	0	1 (0.5%)	0.08
28-day readmission (%)	1191	94 (16.2%)	64 (15.7%)	32 (15.8%)	0.99
6-month mortality (%)	1191	95 (16.4%)	93 (22.8%)	59 (29.2%)	<0.001

Categorical explanatory variables are reported as percentages and continuous explanatory variables are summarized as medians with interquartile ranges in parentheses.

^¶^*p-*values were determined by Fisher’s exact test for categorical variables and Kruskal-Wallis for continuous variables.

^†^Adjusted for age, Charlson comorbidity score (excluding diabetes and age) and eGFR.

Abbreviations: HITH = Hospital in the Home.

In the group without an HbA1c result within the specified time frame, there were no significant differences in hospital outcomes compared to the group with an HbA1c result within the pre-specified time frame except for length of stay (Table S1). Surprisingly, significantly fewer patients in the diabetes group had died within 6 months of their index admission date (16.4%) compared to those with pre-diabetes (22.8%) and those without diabetes (29.2%) (p < 0.001). When adjusted for age, eGFR and Charlson comorbidity score (excluding diabetes and age), the presence of diabetes was associated with a significantly lower risk of 6-month mortality compared to the no diabetes group with an OR of 0.10 (0.03–0.33, p < 0.001) (Table 4). There was a trend toward the presence of pre-diabetes being associated with a lower risk of 6-month mortality compared to no diabetes, when adjusted for age, eGFR and Charlson comorbidity score (excluding diabetes and age), with an OR of 0.34 (0.11–1.12, p = 0.08) but this failed to reach significance. These findings were also supported using HbA1c as a continuous variable – OR 0.50 (0.33–0.76, p = 0.001), that is increasing HbA1c measurements were associated with lower risk of death, following adjustments for age, eGFR and Charlson comorbidity score (excluding diabetes and age).

**Table 4:** Association of diabetes with outcomes.

	Diabetes group compared to no diabetes^†^			Pre-diabetes group compared to no diabetes^†^		
	IRR/OR	p value^*¶*^	95% CI	IRR/OR	p value^*¶*^	95% CI
Length of stay (days, IRR)	0.95	0.42	0.83 to 1.08	0.97	0.64	0.85 to 1.10
ICU admission (OR)	1.27	0.70	0.37 to 4.31	0.72	0.63	0.19 to 2.71
28-day readmission (OR)	0.97	0.91	0.60 to 1.58	0.98	0.94	0.59 to 1.62
6-month mortality (OR)	0.10	<0.001	0.03 to 0.33	0.34	0.08	0.11 to 1.12

Categorical explanatory variables are reported as percentages and continuous explanatory variables are summarized as medians with interquartile ranges in parentheses.

^*¶*^*p-*values were determined by Fisher’s exact test for categorical variables and Kruskal-Wallis for continuous variables.

^†^Adjusted for age, Charlson comorbidity score (excluding diabetes and age) and eGFR.

Abbreviations: HITH = Hospital in the Home; IRR = incidence rate ratio, applicable to continuous variables; OR = odds ratio, applicable to categorical variables.

When further adjusted for age, sex, eGFR, admission cardiac medications (beta blocker, renin-angiotensin blockade and statin use) and atrial fibrillation status, the OR for 6-month mortality in the diabetes group compared to no diabetes remained similar at 0.11 (0.03–0.42, p = 0.001) whilst in the pre-diabetes group, the OR dropped further to 0.22 (0.06–0.85, p = 0.03) and this time reached statistical significance.

### Echocardiographic data

There was no difference between the three groups in terms of left ventricular systolic function with similar proportions from diabetes, pre-diabetes and no diabetes groups with preserved systolic function and mild, moderate or severe systolic dysfunction (Table 5). However, the diabetes group were more likely to have diastolic dysfunction with 73% having an E/E′ ratio of >15 (ratio of mitral peak velocity of early filling (E) to early diastolic mitral annular velocity (E′) which is used to detect left ventricular diastolic dysfunction), indicating elevated filling pressures, compared to 58% in the pre-diabetes group and 62% in the no diabetes group (p = 0.008).

**Table 5:** Transthoracic echocardiogram (TTE) parameters by diabetes status.

	All episodes (n = 1191)	Diabetes (n = 581)	Pre-diabetes (n = 408)	No diabetes (n = 202)	p value^*¶*^
TTE data available	651 (55%)	321 (55%)	230 (56%)	100 (50%)	
LV systolic function	644 (99%)	315 (98%)	229 (99.6%)	100 (100%)	
Normal/preserved	301 (47%)	142 (45%)	113 (49%)	46 (46%)	0.35
Mild dysfunction	99 (15%)	56 (18%)	30 (13%)	13 (13%)	0.27
Moderate dysfunction	118 (18%)	57 (18%)	41 (18%)	20 (20%)	0.99
Severe dysfunction	126 (20%)	60 (19%)	45 (20%)	21 (21%)	0.94
E/e′ septal	541 (83%)	271 (84%)	193 (84%)	77 (77%)	
< 8	12 (0.2%)	6 (2%)	6 (3%)	0	0.22
8–15	171 (32%)	67 (25%)	75 (39%)	29 (38%)	0.01
> 15	358 (66%)	198 (73%)	112 (58%)	48 (62%)	0.008

Transthoracic echocardiogram data obtained from up to 12 months prior to and 2 months post-admission date.

^*¶*^*p-*values were determined by Fisher’s exact test for categorical variables and Kruskal-Wallis for continuous variables.

Abbreviations: LV = left ventricular, E/e′ = ratio of mitral peak velocity of early filling (E) to early diastolic mitral annular velocity (E′).

### Medication use

In order to better estimate pre-admission risk, we used admission cardiac medications in our multivariable analyses.

On admission, 37% of the diabetes group were receiving insulin, 33% were taking metformin and 33% were taking sulphonylureas (Table S2). Only 8% were on a DPP-4 inhibitor – 0.5% were on saxagliptin. 0.2% of patients in the diabetes group in this cohort were on an SGLT-2 inhibitor and a GLP-1 agonist. SGLT-2 inhibitors were only widely available for the last 12 months of data collection.

Those in the diabetes group were significantly more likely to be on evidence-based treatment with regards to cardiac medications – in particular, beta blockers, RAS blockade and statin use (Table 6). In the diabetes group, 67% were on a beta blocker (compared to pre-diabetes 63% and no diabetes 55%, p = 0.01), 61% were on an ACE inhibitor or ARB (compared to pre-diabetes 54% and no diabetes 52%, p = 0.03) and 70% were on a statin (pre-diabetes 52% and no diabetes 44%, p < 0.001). In addition, the diabetes and pre-diabetes groups were more likely to be on an aldosterone antagonist on admission (23% and 24% respectively, compared to no diabetes 12%, p = 0.001) and those in the diabetes group were more likely to be on frusemide (76%, compared to pre-diabetes 65% and no diabetes 61%, p < 0.001). Those in the diabetes group were also more likely to be on other diuretics, dihydropyridine calcium channel blockers, nitrates and antiplatelet medications. There was also a significant difference between the 3 groups for anticoagulant drugs (p < 0.001).

**Table 6:** Admission cardiac medications by diabetes status.

Medication	All episodes (n = 1191)	Diabetes (n = 581)	Pre-diabetes (n = 408)	No diabetes (n = 202)	p value^*¶*^
Beta blocker	758 (64%)	390 (67%)	256 (63%)	112 (55%)	0.011
ACEI	400 (34%)	206 (35%)	131 (32%)	63 (31%)	0.406
ARB	296 (25%)	160 (28%)	91 (22%)	45 (22%)	0.117
ACEI or ARB	683 (57%)	356 (61%)	221 (54%)	106 (52%)	0.026
Frusemide	829 (70%)	442 (76%)	264 (65%)	123 (61%)	< 0.001
Aldosterone antagonist	256 (21%)	132 (23%)	99 (24%)	25 (12%)	0.001
Other diuretic	148 (12%)	83 (14%)	55 (13%)	10 (5%)	0.001
Non-DHP CCB	50 (4%)	22 (4%)	21 (5%)	7 (3%)	0.512
DHP CCB	236 (20%)	135 (23%)	62 (15%)	39 (19%)	0.007
Statin	707 (59%)	406 (70%)	213 (52%)	88 (44%)	< 0.001
Digoxin	122 (10%)	65 (11%)	43 (11%)	14 (7%)	0.219
Ivabradine	4 (0.3%)	3 (0.5%)	0	1 (0.5%)	0.390
Hydralazine	6 (0.5%)	1 (0.2%)	2 (0.5%)	3 (1%)	0.080
Amiodarone	98 (8%)	46 (8%)	32 (8%)	20 (10%)	0.638
Nitrate	290 (24%)	159 (27%)	78 (19%)	53 (26%)	0.008
Anticoagulant	407 (34%)	213 (37%)	150 (37%)	44 (22%)	< 0.001
Antiplatelet	652 (55%)	333 (28%)	200 (49%)	119 (59%)	0.016
Allopurinol	180 (15%)	93 (16%)	61 (15%)	26 (13%)	0.574
NSAID	36 (3%)	12 (2%)	16 (4%)	8 (4%)	0.149
Antidepressant	270 (23%)	126 (22%)	90 (22%)	54 (27%)	0.319

^*¶*^*p-*values were determined by Fisher’s exact test for categorical variables and Kruskal-Wallis for continuous variables.

Abbreviations: ACEI = angiotensin converting enzyme inhibitor, ARB = angiotensin receptor blocker, DHP = dihydropyridine, CCB = calcium channel blocker, NSAID = non-steroidal anti-inflammatory drug.

On discharge, 27% of the diabetes group were taking metformin, 31% were taking a sulphonylurea, 7% were on a DPP-4 inhibitor and 36% were prescribed insulin (Table S2).

The diabetes group were significantly more likely to be prescribed a statin on discharge (67%, compared to 50% pre-diabetes and 39% no diabetes, p < 0.001) and were also more likely to be prescribed a beta blocker compared to both the pre-diabetes and no diabetes groups (72%, compared to 69% pre-diabetes, 64% no diabetes, p = 0.07), although this did not reach statistical significance (Table S3). Both the diabetes and pre-diabetes groups were also more likely to receive an aldosterone antagonist on discharge (30%) compared to the no diabetes group (23%, p = 0.125), although this did not reach statistical significance.

### Atrial fibrillation status

In the diabetes group, atrial fibrillation was documented in the medical history in 52% compared to 58% in those with pre-diabetes and 43% in those without diabetes (p = 0.002).

### Specialist medical follow up

Patients with diabetes were significantly more likely to have specialist follow up in the four-week period following their discharge from hospital (46%) compared to those with pre-diabetes (36%) and those without diabetes (39%) (p = 0.003).

## Discussion

To our surprise and contrary to our initial hypothesis, we demonstrated that the presence of diabetes was associated with a significantly lower risk of 6-month all-cause mortality despite adjustment for age, sex, eGFR, admission cardiac medications and atrial fibrillation status. The finding that more patients with diabetes were prescribed cardioprotective medications on admission and had diastolic dysfunction and closer follow up supports the finding of fewer deaths in the population of heart failure patients with diabetes. Furthermore, there was no association between diabetes status and length of stay, ICU admission or 28-day readmission.

Although there was no difference between the groups in terms of left ventricular systolic function, a significantly larger proportion of the diabetes group had evidence of diastolic dysfunction on transthoracic echocardiogram.

The association between HbA1c, as a marker of glycaemic status and mortality in patients with type 2 diabetes and heart failure remains controversial as previous studies have shown conflicting results. Previous smaller studies have demonstrated a similar paradoxical lower risk of mortality in patients with higher HbA1c levels^16^. Furthermore, in a recent study by Blecker *et al*., higher HbA1c being associated with higher risk of mortality only became significant at HbA1c levels above 12% which could reflect those with relatively new diagnosis of diabetes^5^. As our diabetes group population had relatively well controlled diabetes, perhaps the effect of poor glycaemic control on mortality was not seen in our study. The median HbA1c in the diabetes group in the current study was only 7.0%.

Recently empagliflozin^23^, liraglutide^24^ and semaglutide^25^ have been shown to reduce cardiovascular events in patients with type 2 diabetes and empagliflozin in particular, which has now been approved by the Food and Drug Administration (FDA) for reduction of cardiovascular death in patients with type 2 diabetes and cardiovascular disease, showed lower rates of hospitalisation for heart failure and all-cause mortality compared to placebo^23^. In our study, very few patients were on SGLT-2 inhibitors as they were not widely available until the last 12 months of data collection but increasing use in the future may decrease heart failure hospitalisation and improve outcomes in this group of patients.

The current study findings are likely to be related to the fact that those with diabetes were more likely to be on evidence-based treatment on admission – that is beta blockers, renin-angiotensin blockade and statins, as well as aldosterone antagonists, which have all been demonstrated to have a mortality benefit. Drugs that prevent cardiac remodelling, such as renin-angiotensin system antagonists, may also modulate the severity of heart failure and its consequences. Although we took into consideration and adjusted for the presence of these medications in our analyses, we were unable to account for either dose or duration of treatment. In addition, the no diabetes group were significantly older with a median age of 83 years compared to 79 years in the diabetes group which may contribute to their increased 6-month mortality, however, we did adjust age in the models.

Other possible explanations for this association of diabetes with a lower 6-month mortality rate could include the obesity paradox^26^ as we did not have access to body mass index data and the possibility of non-cardiac death.

The novelty of this study is the feasibility of real time, routine HbA1c testing to identify patients with diabetes to quantify glycaemic status and to identify those individuals who may otherwise have been missed. It also represented a real world experience including patients with a significant comorbidity burden from a large public health service who may frequently be excluded from a clinical trial setting.

We recognise the limitations of the current study, including those inherent to observational studies. Inclusion in the study was determined by ICD-10 coding for heart failure which may be subject to inadvertent miscategorisation. However, we were as comprehensive as possible to include all the ICD codes to identify all the inpatients with heart failure. In addition, we had a relatively short follow up period of 6 months and were unable to obtain cause of death to determine if deaths were cardiac in nature. We also did not have access to weight or body mass index data, blood pressure or LDL cholesterol levels for these episodes. We were also unable to account for medication doses, duration of treatment or medication adherence. In addition, as those with diabetes may be more likely to be admitted to hospital with heart failure compared to those without diabetes, there may be an ascertainment bias.

Nearly half of patients admitted with heart failure had diabetes, with a further one third having pre-diabetes. The presence of diabetes was associated with lower 6-month mortality but was not associated with length of stay, ICU admission or 28-day readmission rates. Pre-diabetes was also associated with a lower 6-month mortality rate compared to the no diabetes group. These findings were also supported using HbA1c as a continuous variable. These observational data are hypothesis generating and are likely to be related to the fact that patients with diabetes are more likely to be prescribed evidence-based cardioprotective medications and were more likely to have diastolic, rather than systolic, dysfunction. Further studies will be needed to delineate the mechanism behind the lower risk of 6-month mortality seen here.

## Methods

In this prospective observational cohort study, routine HbA1c testing was performed using an automated order via Cerner Millennium IT Health Platform on all inpatients aged ≥54 years admitted to a tertiary hospital between July 2013 and January 2016 if they did not have an HbA1c result recorded within the preceding 90 days on the hospital system. All HbA1c results were reported via Cerner and were accessible to patients’ treating doctors^27^.

The routine practice at Austin Health is that a report of inpatients with an HbA1c 8.3% (67 mmol/mol) or greater is generated on a daily basis by the endocrine registrar who reviews the management of these patients. HbA1c results, their interpretation and follow up plans are automatically inserted into patient discharge summaries for patients’ local general practitioners. A second confirmatory test is then recommended in the discharge summary for patients with previously unrecognised diabetes (no diabetes diagnosis documented in the medical record but HbA1c ≥ 6.5% [48 mmol/mol])^27^.

Ethylenediaminetetraacetic acid (EDTA) whole blood was obtained from patients for analysis. HbA1c was measured by turbidimetric inhibition immunoassay (TINIA) on Cobas Integra 800 (Roche Diagnostics, Mannheim, Germany). The assay was standardised to the International Federation of Clinical Chemistry and Laboratory Medicine (IFCC) reference method with a between run co-efficient of variation 2.5% for HbA1c 5.6% (30 mmol/mol) and 1.5% for HbA1c 9.7% (83 mmol/mol)^27^.

Admissions where heart failure was the principal diagnosis were identified using the following ICD-10 codes on review of hospital records: I50.0, I50.1, I50.9, I25.5, I42.0, I42.5, I42.6, I42.7, I42.8, I42.9, I09.9, I11.0, I13.0, I13.2, I43.0, I43.1, I43.2, I43.8, P29.0. All heart failure admission episodes with an HbA1c result within 90 days prior to the admission date or up to 7 days after their admission date were eligible for inclusion in this study. Heart failure admission episodes were then divided into three groups according to their diabetic status using medical records and HbA1c result; (i) ‘diabetes’ if a diagnosis of diabetes had been documented in the medical record regardless of HbA1c result or if the HbA1c result was ≥6.5% (≥48 mmol/mol) without a previous diagnosis of diabetes; (ii) ‘pre-diabetes’ if the HbA1c result was between 5.7% and 6.4% (39 to 46 mmol/mol) with no previous diagnosis of diabetes and (iii) ‘no diabetes’ if the HbA1c result was <5.7% (38 mmol/mol) with no previous diagnosis of diabetes.

Pre-specified baseline demographic data, principal admission diagnosis, clinical characteristics and biochemical laboratory values were extracted from medical records and hospital databases. Estimated glomerular filtration rate (eGFR) was calculated based on the Chronic Kidney Disease Epidemiology Collaboration (CKD-EPI) formulae using extracted data (age, gender and creatinine levels)^28^. Information regarding comorbidities was obtained from medical records to calculate a Charlson comorbidity score to reflect patient comorbidities and their impact. Charlson comorbidity scores were calculated from ICD-10 AM codes using the previously validated ICD-10 adaptation of the Charlson index. This is a validated method of weighting comorbidities depending on their severity and impact on mortality where each chronic condition is assigned a score of 1, 2, 3 or 6 and a higher score indicates greater comorbidity burden. In this study, the Charlson comorbidity score was modified to exclude diabetes and age as these were considered as separate variables^27^.

Medications prescribed on hospital admission and discharge were obtained from hospital records – specifically, the use of metformin, sulphonylureas, dipeptidyl peptidase-4 (DPP-4) inhibitors, sodium-glucose co-transporter-2 (SGLT-2) inhibitors, thiazolidinediones, alpha-glucosidase inhibitors, glucagon-like peptide-1 (GLP-1) agonists, insulin, beta blockers, angiotensin-converting enzyme (ACE) inhibitors or angiotensin receptor blockers (ARB), frusemide, aldosterone antagonists, other diuretics, calcium channel blockers, statins, digoxin, ivabradine, hydralazine, amiodarone, nitrates, anticoagulant agents, antiplatelet agents, allopurinol, non-steroidal anti-inflammatory drugs (NSAID) and antidepressants were recorded.

The presence of atrial fibrillation from either medical notes or electrocardiogram was obtained from medical records. Information regarding whether specialist medical follow up within four weeks of hospital discharge occurred was also obtained from review of hospital records.

Echocardiographic data, specifically left ventricular systolic function, ejection fraction, E/e′ prime (ratio of mitral peak velocity of early filling (E) to early diastolic mitral annular velocity (E′) which is used to detect left ventricular diastolic dysfunction) and right ventricular systolic pressure, from up to 12 months prior to admission and any time afterwards were extracted from the hospital’s echocardiography database. All echocardiograms performed after admission occurred within 2 months of admission date. Normal/preserved systolic function was defined as left ventricular ejection fraction ≥50%, mild dysfunction 40–49%, moderate dysfunction 30–39% and severe dysfunction <30% or as categorised by the reporting cardiologist if a numerical value for ejection fraction was not specified.

Pre-specified hospital admission outcomes were length of stay (LOS), Intensive Care Unit (ICU) admission, requirement for mechanical ventilation, 28-day readmission rate and 6-month mortality rate. 28-day readmission rate was defined as any unplanned repeat admission to the same hospital within a 28-day period after the index admission. 6-month mortality was defined as death from any cause within 6 months from the index admission date.

Mortality data was available if the patient had died during any hospital admission or if the hospital had been notified of their death within the 30-month study period. If a patient had any contact with Austin Health more than 6 months after their index admission date or there was no contact with the health service 6 or more months after admission and a death was not reported to the hospital, they were not considered to be deceased within the 6-month period post-admission. Mortality data was available for 1190 (99.9%) patients.

### Statistical analysis

Continuous explanatory variables were reported as median values with interquartile ranges and analysed using the Kruskal-Wallis or Wilcoxon rank-sum test as appropriate. Categorical explanatory variables were reported as percentages and analysed with Fisher’s exact test. Throughout the 30-month study period, some participants presented multiple times and in these patients, admission episodes were included in the analysis. Multiple admissions from the same patients were clustered in the statistical analysis. Association between diabetes status and outcomes adjusted for age, sex, Charlson score (excluding diabetes and age), eGFR (Chronic Kidney Disease Epidemiology Collaboration equation (CKD-EPI)), medication use (beta blocker, renin-angiotensin system blockade and statin use) and atrial fibrillation status were investigated using an appropriate multivariate regression model -random effects negative binomial regression for length of stay calculations as this outcome is highly skewed, and random effects logistic regression for binary outcomes. Respective effect sizes were reported as adjusted incidence rate ratios (IRR) with corresponding 95% confidence intervals (95% CI) for the length of stay outcome and as odds ratios (ORs) with corresponding 95% CIs for binary outcomes. P-values were calculated from two-tailed tests of statistical significance with the threshold being 5%. All analyses were performed using Stata software version IC 14 (StataCorp, Texas, USA). The study was previously approved by the Austin Health Human Research Ethics Committee (LNR/15/Austin/41) who waived the need for informed consent for a planned practice change agreed to by hospital senior medical staff members as part of the Austin Health Diabetes Discovery Initiative.

### Ethics approval and consent to participate

The study was previously approved by the Austin Health Human Research Ethics Committee (LNR/15/Austin/41) who waived the need for informed consent for a planned practice change agreed to by hospital senior medical staff members as part of the Austin Health Diabetes Discovery Initiative.

## Electronic supplementary material

Dataset 1

## Data Availability

All data generated or analysed during this study are included in this published article (and its Supplementary Information files).
