# Supplementary material for: Routine use of HbA1c amongst inpatients hospitalised with decompensated heart failure and the association of dysglycaemia with outcomes
Source: Sci Rep. 2018 Sep 10;8:13564. doi: 10.1038/s41598-018-31473-8 (PMC6131544; doi:10.1038/s41598-018-31473-8)
Supplement: Supplementary file 1 — Dataset 1 [file 41598_2018_31473_MOESM1_ESM.docx]

**Routine use of HbA1c amongst inpatients hospitalised with decompensated heart failure and the association of dysglycaemia with outcomes**

# Khoo K^1^, Lew J^1,2^, Neef P^3^, Kearney L^4^, Churilov L^5^, Robbins R^6^, Tan A^1^, Hachem M^2^, Owen-Jones L^7^, Lam Q^8^, Hart G^9^, Wilson A^2,10^, Sumithran P^1,2^, Johnson D^3^, Srivastava PM^2,4^, Farouque O^2,4^, Burrell LM^2,3,4^, Zajac JD^1,2^, Ekinci EI^1,2,*^

^1^ Department of Endocrinology, Austin Health, Heidelberg, 3084, Victoria, Australia

^2^ Department of Medicine, Austin Health, The University of Melbourne, Heidelberg, 3084 Victoria, Australia

^3^ Department of General Medicine, Austin Health, Heidelberg, 3084, Victoria, Australia

^4^ Department of Cardiology, Austin Health, Heidelberg, 3084, Victoria, Australia

^5^ The Florey Institute of Neuroscience and Mental Health, Heidelberg, 3084, Victoria, Australia

^6^ Department of Strategy, Quality & Service Redesign, Austin Health, Heidelberg, 3084, Victoria, Australia

^7^ Austin Centre for Applied Clinical Informatics, Austin Health, Heidelberg, 3084, Victoria, Australia

^8^ Department of Pathology, Austin Health, Heidelberg, 3084, Victoria, Australia

^9^ Department of Intensive Care, Austin Health, Heidelberg, 3084, Victoria, Australia

^10^ St Vincent’s Hospital Melbourne, Fitzroy, 3065, Victoria, Australia

*elif.ekinci@unimelb.edu.au

**Supplementary data**

**Table S1: Patient outcomes – Heart failure admissions without HbA1c**

| **Outcome** | **N (episodes)** | **No diabetes** | **p value*^¶^*** |
| --- | --- | --- | --- |
| Length of stay (days, excluding HITH) | 270 | 4 (2, 7) | 0.003 |
| ICU admission (%) | 270 | 13 (4.8%) | 0.49 |
| Mechanical ventilation (%) | 270 | 2 (0.7%) | 0.68 |
| 28-day readmission (%) | 270 | 56 (20.7%) | 0.07 |
| 6-month mortality (%) | 270 | 55 (20.4%) | 0.93 |

*^¶^ p-value compared to group with an HbA1c within the pre-specified time frame. P-values were determined by Fisher’s exact test for categorical variables and Wilcoxon rank-sum test for continuous variables.*

**Table S2: Admission and discharge diabetes medications in the diabetes group**

| **Medication** | **Admission**  **(n = 581)** | **Discharge**  **(n = 581)** |
| --- | --- | --- |
| Metformin | 190 (16%) | 156 (13%) |
| Sulphonylurea | 190 (16%) | 180 (15%) |
| Saxagliptin | 3 (0.3%) | 3 (0.3%) |
| Sitagliptin | 18 (2%) | 14 (1%) |
| Linagliptin | 16 (1%) | 15 (1%) |
| Vildagliptin | 7 (0.6%) | 7 (0.6%) |
| Alogliptin | 0 | 0 |
| Any DPP-4 inhibitor | 44 (4%) | 39 (3%) |
| Dapagliflozin | 1 (0.08%) | 1 (0.1%) |
| Empagliflozin | 0 | 0 |
| Thiazolidinedione | 6 (0.5%) | 1 (0.1%) |
| Alpha-glucosidase inhibitor | 6 (0.5%) | 5 (0.4%) |
| Any OHA | 310 (26%) | 287 (24%) |
| GLP-1 agonist | 1 (0.08%) | 1 (0.1%) |
| Insulin | 217 (18%) | 207 (17%) |
| Diet | 32 (3%) | 28 (2%) |

**Table S3: Discharge cardiac medications by diabetes status**

| **Medication** | **All episodes**  **(n = 1191)** | **Diabetes**  **(n = 581)** | **Pre-diabetes**  **(n = 408)** | **No diabetes**  **(n = 202)** | **p value*^¶^*** |
| --- | --- | --- | --- | --- | --- |
| **Beta blocker** | 829 (70%) | 420 (72%) | 280 (69%) | 129 (64%) | 0.070 |
| **ACEI** | 444 (37%) | 207 (36%) | 163 (40%) | 74 (37%) | 0.377 |
| **ARB** | 209 (18%) | 112 (19%) | 60 (15%) | 37 (18%) | 0.167 |
| **ACEI or ARB** | 650 (55%) | 317 (55%) | 223 (55%) | 110 (54%) | 1.000 |
| **Frusemide** | 1007 (85%) | 502 (86%) | 345 (85%) | 160 (79%) | 0.057 |
| **Aldosterone antagonist** | 341 (29%) | 174 (30%) | 121 (30%) | 46 (23%) | 0.125 |
| **Other diuretic** | 146 (12%) | 95 (16%) | 42 (10%) | 9 (4%) | <0.001 |
| **Non-DHP CCB** | 25 (2%) | 14 (2%) | 7 (2%) | 4 (2%) | 0.739 |
| **DHP CCB** | 191 (16%) | 118 (20%) | 44 (11%) | 29 (14%) | <0.001 |
| **Statin** | 673 (57%) | 388 (67%) | 206 (50%) | 79 (39%) | <0.001 |
| **Digoxin** | 131 (11%) | 67 (6%) | 49 (12%) | 15 (7%) | 0.191 |
| **Ivabradine** | 6 (0.5%) | 3 (0.5%) | 2 (0.5%) | 1 (0.5%) | 1 |
| **Hydralazine** | 3 (0.3%) | 1 (0.2%) | 1 (0.2%) | 1 (0.5%) | 0.583 |
| **Amiodarone** | 108 (9%) | 50 (9%) | 36 (9%) | 22 (11%) | 0.602 |
| **Nitrate** | 276 (23%) | 152 (26%) | 75 (18%) | 49 (24%) | 0.014 |
| **Anticoagulant** | 411 (35%) | 207 (36%) | 152 (37%) | 52 (26%) | 0.013 |
| **Antiplatelet** | 644 (54%) | 335 (58%) | 195 (48%) | 114 (56%) | 0.007 |
| **Allopurinol** | 171 (14%) | 97 (17%) | 55 (13%) | 19 (9%) | 0.030 |
| **NSAID** | 7 (0.6%) | 2 (0.3%) | 3 (0.7%) | 2 (1%) | 0.355 |
| **Antidepressant** | 254 (21%) | 124 (21%) | 83 (20%) | 47 (23%) | 0.702 |
